# miR-125b targets erythropoietin and its receptor and their expression correlates with metastatic potential and ERBB2/HER2 expression

**DOI:** 10.1186/1476-4598-12-130

**Published:** 2013-10-28

**Authors:** Manuela Ferracin, Cristian Bassi, Massimo Pedriali, Sara Pagotto, Lucilla D’Abundo, Barbara Zagatti, Fabio Corrà, Gentian Musa, Elisa Callegari, Laura Lupini, Stefano Volpato, Patrizia Querzoli, Massimo Negrini

**Affiliations:** 1 Department of Morphology, Surgery and Experimental Medicine, University of Ferrara, Ferrara, Italy; 2 Laboratory for Technologies of Advanced Therapies (LTTA), University of Ferrara, Via Fossato di Mortara 70, Ferrara 44121, Italy; 3 Non-coding RNA and Cancer Unit, Aging Research Center (Ce.S.I.), Fondazione Università "G. D'Annunzio", Chieti, Italy; 4 Department of Medical Sciences, University of Ferrara, Ferrara, Italy

**Keywords:** miR-125b, MicroRNA, Erythropoietin, Breast cancer, ERBB2

## Abstract

**Background:**

The microRNA 125b is a double-faced gene expression regulator described both as a tumor suppressor gene (in solid tumors) and an oncogene (in hematologic malignancies). In human breast cancer, it is one of the most down-regulated miRNAs and is able to modulate ERBB2/3 expression. Here, we investigated its targets in breast cancer cell lines after miRNA-mimic transfection. We examined the interactions of the validated targets with ERBB2 oncogene and the correlation of miR-125b expression with clinical variables.

**Methods:**

MiR-125b possible targets were identified after transfecting a miRNA-mimic in MCF7 cell line and analyzing gene expression modifications with Agilent microarrays and Sylamer bioinformatic tool. Erythropoietin (EPO) and its receptor (EPOR) were validated as targets of miR-125b by luciferase assay and their expression was assessed by RT-qPCR in 42 breast cancers and 13 normal samples. The molecular talk between EPOR and ERBB2 transcripts, through miR-125b, was explored transfecting MDA-MD-453 and MDA-MB-157 with ERBB2 RNA and using RT-qPCR.

**Results:**

We identified a panel of genes down-regulated after miR-125b transfection and putative targets of miR-125b. Among them, we validated erythropoietin (EPO) and its receptor (EPOR) - frequently overexpressed in breast cancer - as true targets of miR-125b. Moreover, we explored possible correlations with clinical variables and we found a down-regulation of miR-125b in metastatic breast cancers and a significant positive correlation between EPOR and ERBB2/HER2 levels, that are both targets of miR-125b and function as competing endogenous RNAs (ceRNAs).

**Conclusions:**

Taken together our results show a mechanism for EPO/EPOR and ERBB2 co-regulation in breast cancer and confirm the importance of miR-125b in controlling clinically-relevant cancer features.

## Background

MicroRNA (miRNA) expression deregulation in human breast cancer was one of the first to be described worldwide [1]. Among the most down-regulated miRNAs in breast cancer compared to normal mammary tissue there was microRNA 125b (miR-125b), suggesting a possible role for this miRNA as a tumor suppressor gene. Indeed, its expression was found to be reduced or completely lost in a variety of solid cancers including lung [2,3], hepatic [4], thyroid [5], ovarian [6], cervical [7] cancer, melanoma [8] and neuroblastoma [9] with the exception of gastric [10] and pancreatic [11] cancers in which it is up-regulated. A germline mutation in hsa-miR-125a locus, that lead to reduced levels of miR-125a (whose mature form is equal to miR-125b) has been described in breast cancer patients [12].

More recently, a true double-faced role for miR-125b in malignant transformation emerged, with the identification of the oncogenic properties of miR-125b overexpression in hematological malignancies [13-16]. The overexpression of miR-125b in hematological neoplasia is often due to translocations involving *hsa-miR-125b-1* locus on chromosome 11q24: t(2;11) in myelodysplastic syndrome and t(11;14) in acute lymphoblastic leukemia.

It seems therefore evident that miR-125b is an intriguing miRNA with multiple functions as a regulator of cell proliferation, differentiation and apoptosis that strictly depends on the cellular context. Among the most relevant targets in solid tumors there are the anti-apoptotic and pro-proliferative proteins Bcl-2 [17], c-jun [8], c-raf [18], p53 [19], ERBB2 [20], ERBB3 [20], MUC1 [21], PIK3CD [22].

In breast cancer, miR-125b down-regulation is an early event in cancer progression [23] and it is partially due to its epigenetic regulation through promoter methylation [24]. A reduced expression of this miRNA in people that carry a mutation in *ATM* gene has been described [25]. Reduced levels of miR-125b have been linked to an increased metastatic capability of breast cancer cells [24] and to an increase in cell motility in vitro [26]. Moreover, a single nucleotide polymorphism in MRE (miRNA response element) for miR-125b of BMPR1B gene has been linked to the risk of developing breast cancer [27].

In this study, we describe erythropoietin (EPO) and erythropoietin receptor (EPOR) as novel targets of miR-125b and we tested the hypothesis of an association between miRNA/targets expression and clinical outcomes in breast cancer. Moreover, we discovered a cross-talk between EPOR and ERBB2/HER2 based on their acting as decoys for miR-125b.

## Results

### Identification of miR-125b target genes in MCF7 cell line and their functional annotation

To identify possible targets of human miR-125b, we transfected MCF7 breast cancer cell line with a miR-125b mimic oligonucleotide (Ambion) or a negative control oligonucleotide (#2, Ambion) and we collected total RNA at 24 and 48 hours after transfection. Global gene expression changes were examined using microarray technology (Agilent Whole Human Genome microarray). We applied Sylamer algorithm [28], that searches for enriched sequences in 3′UTR of genes, to the list of genes down-modulated by miR-125b both at 24 and 48 hours after transfection. As expected, miR-125b seed sequence (7- 8-mer) was significantly enriched in down-regulated transcripts (Additional file 1: Figure S1). We found a list of 315 genes (Additional file 2: Table S1) whose 3′UTR contains at least 1 region complementary to the seed sequence of miR-125b (7-mers (C)TCAGGG(A) and 8-mer CTCAGGGA) and whose expression is reduced after transfection, thereby supporting a direct role of the miRNA in causing mRNA down-regulation.

**Figure 1:**
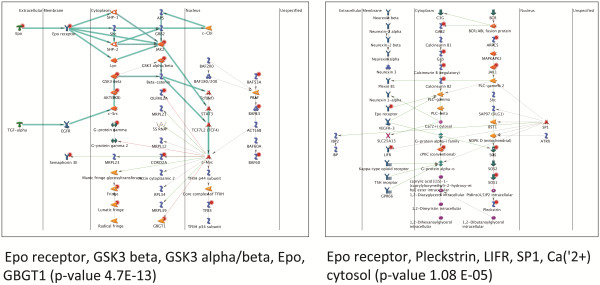
**Network analysis of genes modulated by miR-125b and putative targets of miR-125b.** Network analysis, performed on genes down-modulated after miR-125b transfection in MCF7 and containing a MRE in their 3′UTR, revealed two enriched network of genes relying on EPO/EPOR signaling and involving several genes that are targeted by miR-125b (red circles).

We analyzed the list of genes down-regulated after miR-125b transfection and containing a potential miRNA response element (MRE) with Metacore (GeneGO) software and we found the significantly enriched pathways (Table 1). Among them, there are two pathways concerning Erythopoietin (EPO) signaling and the Metacore network analysis revealed that EPO signaling is deeply influenced by miRNA transfection (Figure 1). Indeed, both EPO and EPzOR (Erythopoietin receptor) are putative targets of miR-125b (Additional file 3: Figure S2) and their downregulation after miR-125b transfection was validated by quantitative RT-PCR (RT-qPCR), both at 24 and 48 hours (Additional file 4: Figure S3).

**Table 1:** Pathways significantly enriched in the list of genes down-regulated by miR-125b and with a MRE in 3'UTR (source: GeneGO Metacore)

**#**	**Maps**	**pValue**	**Ratio**	
1	Immune response_CD28 signaling	2.304E-04	5	54
2	Development_EPO-induced PI3K/AKT pathway and Ca(2+) influx	9.912E-04	4	43
3	Neurophysiological process_Melatonin signaling	9.912E-04	4	43
4	Development_A2A receptor signaling	9.912E-04	4	43
5	Development_EPO-induced MAPK pathway	1.178E-03	4	45
6	Development_WNT5A signaling	1.280E-03	4	46
7	Neurophysiological process_NMDA-dependent postsynaptic long-term potentiation in CA1 hippocampal neurons	1.417E-03	5	80
8	Nicotine signaling in dopaminergic neurons, Pt. 1 - cell body	1.502E-03	4	48
9	Development_A2B receptor: action via G-protein alpha s	1.750E-03	4	50
10	Signal transduction_PKA signaling	1.884E-03	4	51
11	Immune response_HSP60 and HSP70/ TLR signaling pathway	2.329E-03	4	54
12	Cell adhesion_Gap junctions	3.608E-03	3	30
13	Development_EGFR signaling pathway	4.086E-03	4	63
14	Oxidative stress_Role of ASK1 under oxidative stress	5.161E-03	3	34
15	Reproduction_GnRH signaling	6.576E-03	4	72
16	Development_Mu-type opioid receptor signaling	7.062E-03	3	38
17	Signal transduction_cAMP signaling	7.062E-03	3	38
18	Transcription_P53 signaling pathway	7.594E-03	3	39
19	Development_PACAP signaling in neural cells	7.594E-03	3	39
20	Apoptosis and survival_Lymphotoxin-beta receptor signaling	9.328E-03	3	42
21	Nicotine signaling in dopaminergic neurons, Pt. 2 - axon terminal	9.953E-03	3	43
22	Development_S1P1 signaling pathway	1.060E-02	3	44
23	Neurophysiological process_Glutamate regulation of Dopamine D1A receptor signaling	1.128E-02	3	45
24	Transcription_Androgen Receptor nuclear signaling	1.128E-02	3	45
25	Development_Thrombopoietin-regulated cell processes	1.128E-02	3	45
26	Development_GDNF family signaling	1.197E-02	3	46
27	Neurophysiological process_Dopamine D2 receptor signaling in CNS	1.269E-02	3	47
28	Development_TGF-beta-dependent induction of EMT via MAPK	1.269E-02	3	47
29	Development_HGF signaling pathway	1.269E-02	3	47
30	Immune response_Histamine H1 receptor signaling in immune response	1.344E-02	3	48
31	Immune response_Lectin induced complement pathway	1.421E-02	3	49
32	Development_A3 receptor signaling	1.421E-02	3	49
33	Immune response_Histamine signaling in dendritic cells	1.501E-02	3	50
34	Mucin expression in CF via TLRs, EGFR signaling pathways	1.501E-02	3	50
35	Immune response_NFAT in immune response	1.583E-02	3	51
36	Development_IGF-1 receptor signaling	1.667E-02	3	52
37	Signal transduction_Activation of PKC via G-Protein coupled receptor	1.667E-02	3	52
38	Cell adhesion_ECM remodeling	1.667E-02	3	52
39	Immune response_Classical complement pathway	1.667E-02	3	52
40	Development_Endothelin-1/EDNRA signaling	1.754E-02	3	53
41	Membrane-bound ESR1: interaction with G-proteins signaling	1.844E-02	3	54
42	Development_FGFR signaling pathway	1.844E-02	3	54
43	Immune response_Fc epsilon RI pathway	1.936E-02	3	55
44	Regulation of lipid metabolism_Insulin regulation of glycogen metabolism	2.030E-02	3	56
45	Cytoskeleton remodeling_FAK signaling	2.127E-02	3	57
46	Immune response_Immunological synapse formation	2.329E-02	3	59
47	Development_EGFR signaling via PIP3	2.351E-02	2	23
48	Development_Thyroliberin signaling	2.540E-02	3	61
49	Development_Gastrin in cell growth and proliferation	2.650E-02	3	62
50	G-protein signaling_K-RAS regulation pathway	2.750E-02	2	25

### EPO and EPOR are target of miR-125b

**Figure 2:**
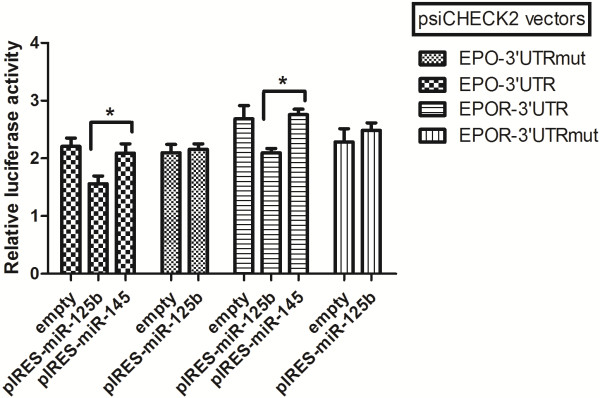
**EPO and EPOR are targets of miR-125b.** Luciferase reporter assays was performed with psiCHECK2 reporter vector after placing Renilla luciferase under the control of the EPO/EPOR 3′UTR. A not-targeting miRNA (miR-145), the empty pIRESneo2 vector and the mutated version of psiCHECK2 vectors were used as controls. The Renilla luciferase values were normalized for transfection with Firefly luciferase activity and data are presented relative to the vector control. The mean ± s.d. of three independent experiments in two different cell lines is shown. *P <0.05.

Transfection experiments were performed in MCF7 and HEK-293 cell lines to validate EPO and EPOR as targets of miR-125b. EPO and EPOR 3′UTRs were cloned downstream Renilla luciferase gene in psiCHECK-2 reporter vector. This vector contains also Firefly luciferase gene that can be used as a reference in Dual-Luciferase Reporter Assay (Promega). These vectors were transfected in two different cell lines together with a vector expressing miR-125b (pIRESneo2- miR125b), while the empty pIRESneo2 vector, pIRES-neo2 vector expressing miR-145 (that does not target EPO/EPOR 3′UTRs) and the mutated version of psiCHECK-2 + 3′UTR plasmids were used as negative controls. The luciferase activity of the constructs containing EPO and EPOR-3′UTR was suppressed of ~30% (p < .05) following ectopic expression of miR-125b (Figure 2) demonstrating that EPO/EPOR expression is under the control of miR-125b.

### miR-125b and EPO/EPOR expression is inversely correlated in breast cancer

**Figure 3:**
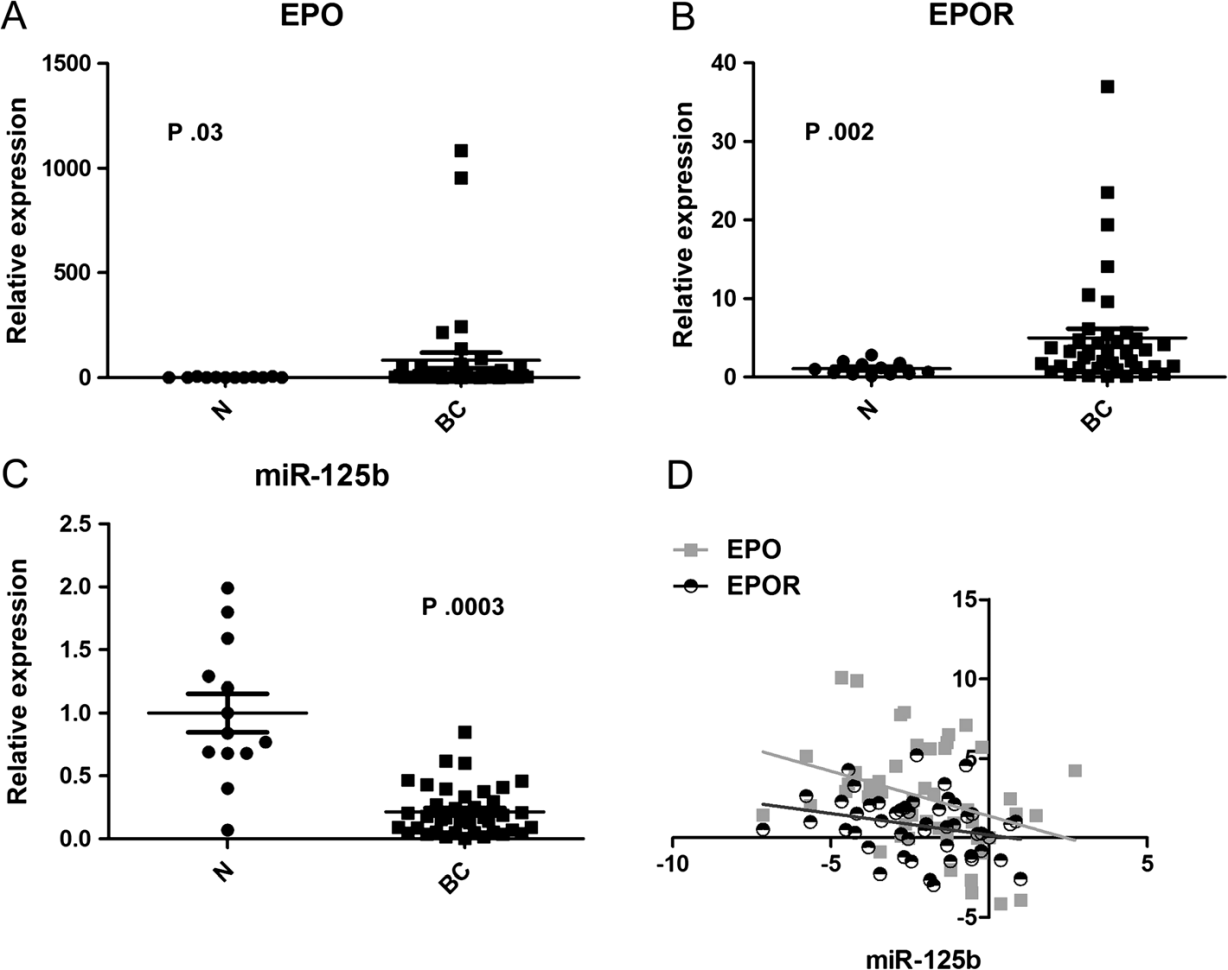
**Relative expression of miR-125b, EPO and EPOR in human breast cancer.** The expression levels of EPO **(A)**, EPOR **(B)** and miR-125b **(C)** was measured in 42 breast cancers and 13 normal breasts by RT-qPCR. RNU6 and 18S were used as reference genes for miRNA and genes respectively. 2-deltaCq method was used for normalized relative expression calculation. **D)** A statistically significant inverse correlation between miR-125b and EPO and EPOR expression (P < .05 for both genes) was observed by using two-tailed Spearman’s test (log2 data).

We validated the opposite modulation of miR-125b, EPO and EPOR by RT-qPCR in a cohort of 42 breast cancer patients and 13 normal samples collected at the University of Ferrara (Figure 3A-C). We found a significant down-regulation of miR-125b and an up-regulation of EPO and EPOR genes (p = 0.0003, 0.03 and 0.002, respectively; unpaired t-test with Welch’s correction). Likewise, a significant inverse correlation between miR-125b and EPO (p = 0.0037) and EPOR (p = 0.03) genes was found (Spearman correlation, r = -039; -0.31, Figure 3D). These observations confirm the role of miR-125b in controlling the up-regulation of Erythropoietin and Erythropoietin receptor in breast cancer.

### Correlation of miR-125b and EPOR expression with metastatic potential and ERBB2 expression

**Figure 4:**
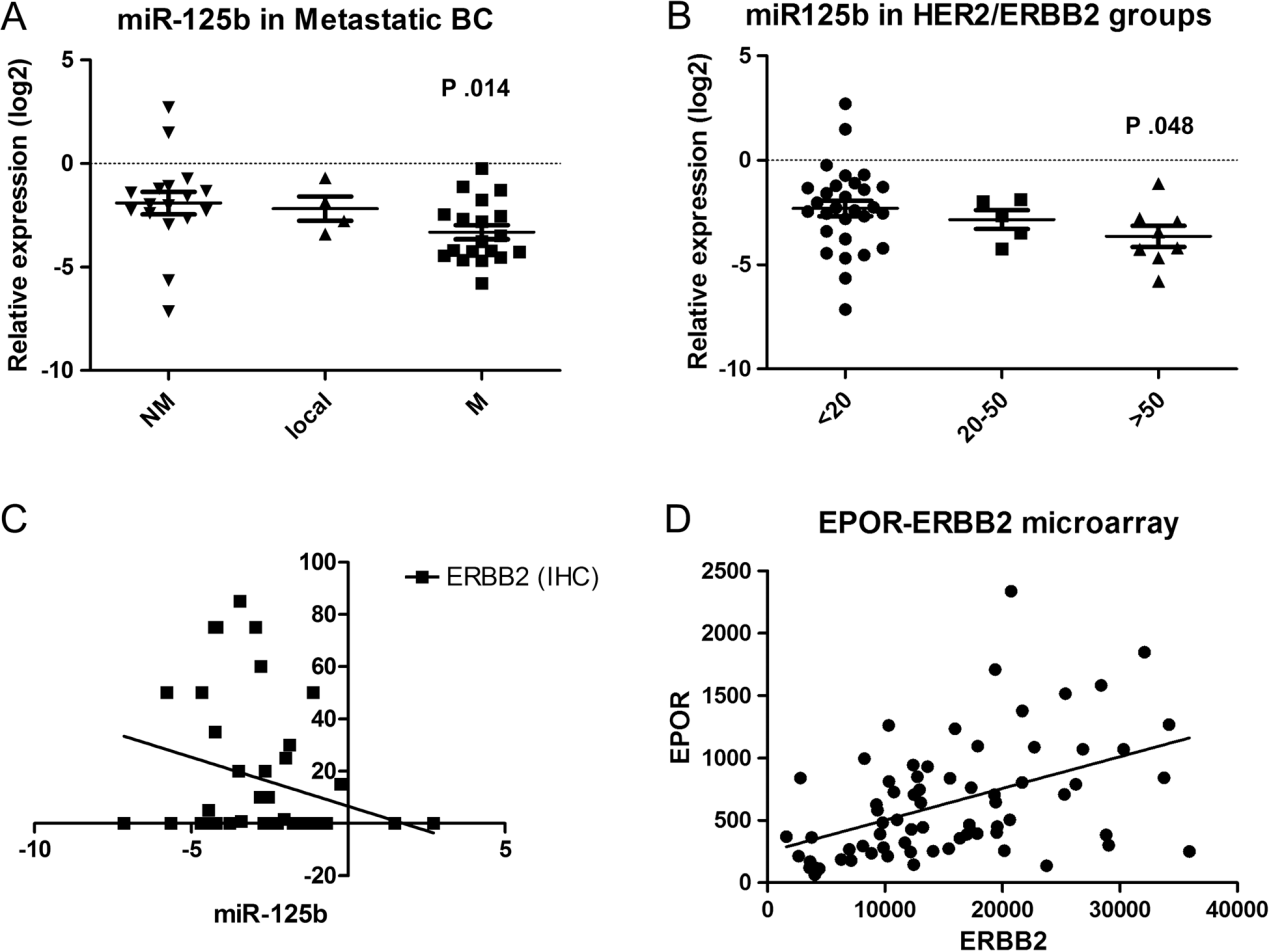
**Correlation of miR-125b and EPOR with metastasis and ERBB2. A)** Expression of miR-125b (normalized on RNU6 and log2 transformed) in not metastatic (NM), locally recurrent (local) and metastatic (M) breast cancer, detected by RT-qPCR. A significant two-tailed Mann Whitney test) down-regulation in metastatic BC can be observed. **B)** MiR-125b levels are significantly (p = 0.048, two-tailed Mann Whitney test) down-regulated in ERBB2 strongly positive breast cancers (IHC positive cells > 50%) **C)** and display an inverse correlation (Spearman r = 0.31, p = 0.04). **D)** Microarray data from 69 breast cancer without HER-2 amplification shows a highly concordant expression (Spearman r = 0.54, p < 0.0001) of ERBB2/HER2 and EPOR levels.

To evaluate the significance of miR-125b, EPO and EPOR expression in breast cancer, we examined their expression levels in breast cancer patients, grouped according to the following clinical-pathological variables: metastatic recurrence, p53 mutation, grade, stage, lymphnode invasion, proliferative index, estrogen and progesterone receptors positivity, ERBB2 overexpression. We found a significant down-regulation of miR-125b levels in metastatic breast cancers (Figure 4A) compared to not metastatic tumors (p = 0.014, two-tailed Mann Whitney test). A reduced expression of miR-125b can be observed also in locally recurrent breast cancers, although only four samples were available. EPO and EPOR levels were only slightly increased in metastatic cancers without reaching significance (data not shown).

ERBB2 (HER2) gene is a validated target of miR-125b [20]. To confirm the regulation of ERBB2 by miR-125b we examined the miRNA levels in breast cancers strongly positive (positive cells > 50%) intermediately positive (positive cells = 20-50%) and weakly positive or negative (positive cells < 20%) for ERBB2 (Figure 4B). As expected, miR-125b expression is significantly reduced in strongly positive breast cancer (p = 0.04, two-tailed Mann Whitney test). A significant inverse correlation was found between miR-125b expression levels and ERBB2 protein levels as well (Spearman correlation, r = -031; p = 0.04; Figure 4C).

Since an extensive co-expression of ERBB2 and EPOR has been described in breast cancer [29] and ERBB2 protein levels correlates with that of ERBB2 mRNA [30,31], we evaluated the relationship between ERBB2 and EPOR using gene expression data from 69 breast cancers with low and intermediate levels of ERBB2 (data from proprietary microarray experiments, average or two probes for each gene). Normalized expression data are available in Additional file 5: Table S2. We found a significant positive correlation between EPOR and ERBB2 expression levels (Spearman correlation, r = 0.54; two-tailed p-value < 0.0001; Figure 4D). This result confirms the data obtained from qPCR and IHC data that were previously described.

No significant association was found between miRNA/gene expression levels and grade, stage, lymphnode invasion, proliferative index, estrogen and progesterone receptors positivity.

### EPOR and ERBB2 act as decoys for miR-125b

**Figure 5:**
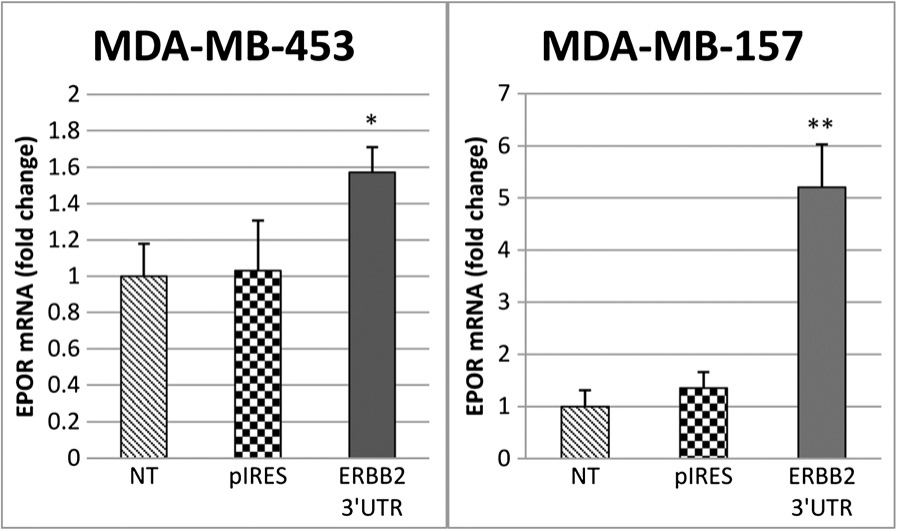
**ERBB2 3′UTR acts as a decoy for miR-125b.** MDA-MD-453 and MDA-MB-157 (HER2+, EPOR+, miR-125b+) breast cancer cell lines were transfected with pIRES-ERBB2-3’UTR vector and show increased EPOR mRNA levels at 24 hours. Data were obtained from RT-qPCR experiments performed in triplicate. 18S was used as reference gene.

We hypothesized that ERBB2 and EPOR could act as competing endogenous RNAs (ceRNAs), influencing their respective levels using miR-125b response elements present in their 3′ UTR. Since ERBB2 is frequently amplified or overexpressed in breast cancer [32], we used its 3′UTR as a driver for EPOR levels through miR-125b decoy. We selected two breast cancer cell lines (MDA-MB-453 and MDA-MB-157) characterized by low-intermediate levels of endogenous ERBB2 (Additional file 6: Figure S4), moderate-high EPOR expression [29] and low levels of endogenous miR-125b (if compared to normal breast, data not shown). We transfected both cell lines with pIRES-ERBB2-3UTR plasmid that express ERBB2 3′UTR. We used the empty plasmid as well as not-transfected cells as controls. As represented in Figure 5, we observed a significant increase of EPOR levels (two-tailed unpaired t-test) 24 hours after the transfection in both cell lines, using RT-qPCR assay.

## Discussion

Among the genes modulated after miR-125b transfection and putative targets of the same miRNA, we focused our attention on erythropoietin and its receptor. Erythropoietin (EPO) has long been known as the cytokine that regulates differentiation and survival of erythroid cells. Recombinant human EPO (rHuEPO) has been used in cancer patients for the treatment of chemotherapy-induced anemia for two decades. However, recent studies revealed a more pleiotropic role for this cytokine and several clinical trial reported an increase mortality in cancer patients treated with erythropoiesis stimulating agents (ESAs) compared to control groups [33].

EPO receptor (EPOR) is a member of type I cytokine receptor family and the binding with EPO ligand activates many important pathways of the cell, such as JAK2/STAT5, MAPK(ERK) and PI3K/AKT. EPO receptor is expressed in a variety of non-hematopoietic human tissues in which the EPO-EPOR signaling works to increase survival and to protect cells from injury [34]. In 2001, Acs and coworkers first described an increased expression of EPO-EPOR in breast cancer samples and cell lines, hypothesizing a paracrine loop able to sustain cell proliferation [35]. Now, we know that EPO-EPOR signaling is active in a variety of solid tumors [33], that EPO-EPOR expression is particularly increased in hypoxic regions [36] and can influence cancer resistance to pharmacological treatments [29,37]. Specifically, it has been demonstrated that EPOR activation is able to antagonize trastuzumab therapeutic effect in vitro, thanks to the activation of a proliferative signaling pathway overlapping with ERBB2 pathway [29] and that EPOR-positive breast cancer patients display a reduced response to tamoxifen [37].

In this work, we confirmed the down-regulation of miR-125b and the up-regulation of EPO and EPOR in a large panel of breast cancers. Moreover, we describe one of the mechanisms that regulate EPO-EPOR expression in breast cancer that is miRNA-dependent. Indeed, both EPO and EPOR 3′UTRs have at least one target sequence for miR-125b that responded to a miR-125b mimic transfection in the context of breast cancer cells. Therefore, we speculated that the described overexpression of EPO-EPOR in several types of carcinoma could be at least in part due to the concomitant loss of miR-125b.

In this study we further explored the correlation between miR-125b, EPO, EPOR and clinical-pathological variables. We found that miR-125b levels are reduced in metastatic breast cancer, that is congruent with its role as a tumor suppressor miRNA in this cancer type and with published data [18,24,38]. We found also a reduced expression of miR-125b in breast cancers strongly positive for ERBB2, that is coherent with its being a target of the miRNA. Since EPOR and ERBB2 are both target of the tumor suppressor miR-125b, we searched for a correlation between EPOR and ERBB2 expression in an independent cohort of breast cancers. We found a positive correlation between the levels of the two receptors, suggesting the existence of mechanisms of co-regulation and possibly functional cooperation, especially in breast cancers where ERBB2/HER2 increased levels cannot alone sustain/explain the malignant behavior. Indeed, both genes are regulated by miR-125b and are able to activate the same molecular pathways.

By examining the ability of ERBB2 3′UTR to function as a decoy of miR-125b, we were indeed able to induce an increase in EPOR levels, probably by reducing the amount of “free” cellular miR-125b, thereby revealing a mechanism for their positive correlation. Notably, it has also been recently demonstrated that miR-125b can target PIK3CD [22], a key mediator of both EPOR and ERBB2 downstream pathways. The reduced levels of miR-125b in breast cancer cells suggests that both ERBB2 and EPOR could become up-regulated and actively cooperate to increase cell proliferation and reduce the apoptotic rate in cancer cells. The active role of these receptors in promoting breast cancers needs to be further studied, to better understand their role and possible cooperation in sustaining the growth of breast cancer cells. Indeed, a recent report indicated that HER2 negative tumors may still benefit of anti HER2 therapies [39].

## Conclusions

In conclusion, we demonstrated that the tumor-suppressive miR-125b is able to modulate erythropoietin/ erythropoietin receptor axis in breast cancer and display a negative correlation with its metastatic potential. The erythropoietin receptor has a positive correlation with the epidermal growth factor receptor b2, that is explained by their acting as decoy molecules for miR-125b. Finally, our study indicates that a special attention should be given to miR-125b whose role as tumor-suppressor in several solid cancers is becoming strong. miR-125b is definitely a significant cancer-associated miRNA and the comprehension of its mechanism of action in specific cellular contexts is important to evaluate its use either as a therapeutic molecule or as therapeutic target.

## Methods

### Patients

Breast cancer and normal breast tissues were anonymously collected at the University of Ferrara (Italy) and extensively characterized for clinical and bio-pathologic status by two expert pathologists (P.Q. and M.P.). Clinico-pathological information concerning metastasis development, ERBB2/HER2 expression, p53 mutation, grade, stage, lymphnode invasion, proliferative index, estrogen and progesterone receptors positivity was available for all tumor samples and determined as previously described [1]. Total RNA isolation was performed with Trizol (Invitrogen) according to the manufacturer’s instructions.

### Cell lines and transfection

MDA-MB-453, MDA-MB-157, MCF7 and HEK-293 cell lines were obtained from the ATCC and were cultured with ISCOVE’s Modified Dulbecco’s Medium (Lonza BioWhittaker) with 10% fetal bovine serum (FBS) and gentamicin. For microarray experiments, MCF7 cell line was transfected with 100 nM of miR-125b (Ambion) or Negative Control#2 (Ambion); total RNA was extracted at 24hs and 48 hs after transfection by adding Trizol Reagent (Invitrogen) according to manufacturer’s procedure. The day before transfection cells were seeded in antibiotic-free media. Transfection of miRNAs was carried out using Lipofectamine 2000 (Life Technologies) in accordance with manufacturer’s procedure.

### Plasmids

The human EPO and EPOR 3′-UTR target sites were amplified by PCR using the following primers: EPO-3UTR_F: 5′- ATCTCGAGCTCCCTCACCAACATTGCTT-3′; EPO-3UTR_R: 5′- ATGTTTAAACGTCTTCATGGTTCCCACCAC-3′; EPOR-3UTR_F: 5′- ATCTCGAGCCAGCTATGTGGCTTGCTCT-3′; EPOR-3UTR_R: 5′- ATGTTTAAACACTGCAAGGTTGTGGTTTCC-3′ and cloned downstream of the Renilla luciferase gene in psiCHECK-2 vector (Promega). Mutated 3′UTRs, through the deletion of the miR-125b seeds inside EPO and EPOR 3′UTRs, were generated using gBlocks Gene Fragments (IDT). These vectors - psiCHECK2-EPO, psiCHECK2-EPOR and their mutated versions - were used for transfection into MCF7 and HEK-293 cells. Vector expressing miR-125b and miR-145 (used as negative control) were obtained after cloning miR-125b and miR-145 genomic sequences in pIRESneo2 expression vector (Clontech). The level of miR-125b expression in transfected cells was assayed by RT-qPCR (TaqMan MicroRNA Assays, Life Technologies) 24hs and 48 hs after transfection (data not shown). The 3′-UTR of ERBB2 was amplified by PCR from the cDNA of T47D and SK-BR3 cell lines (HER2 positive) and cloned into XbaI and EcoRI sites of pIRESneo2 expression plasmid.

### Luciferase assay

MCF7 and HEK-293 cell lines were seeded in 24-well plates at a cellular concentration of 40,000 and 100,000 cells/well respectively. Cells were transfected with 400 ngs of psiCHECK2-EPO or equimolar amounts of psiCHECK2-EPOR or mutant control vectors together with equimolar amounts of pIRESneo2-miR125b or pIRESneo2-control (miR-145). Transfection was performed using Lipofectamine 2000 and OPTI-MEM I Reduced Serum Medium (GIBCO). Twenty-four hours after transfection Firefly and Renilla luciferase activity were measured using the Dual-Luciferase Reporter Assay (Promega). Each transfection was repeated in triplicate.

### Sponge experiment

MDA-MB-453 (7x10^5^/well) and MDA-MB-157 (2x10^5^/well) were seeded in 6-well plates in antibiotic-free media. The following day, they were transfected with 2.5 μg of pIRESneo2 or pIRES-ERBB2-3UTR using Lipofectamine LTX (Life Technologies) and OPTI-MEM I Reduced Serum Medium according to the manufacturer’s recommendations. 24 hours later, cells were collected using Trizol Reagent for RNA extraction. The primers used for PCR amplification of ERBB2 3′UTR were ERBB2-3UTR_F: 5′-CAGAATTCTGCCAGTGTGAACCAGAAG-3′ ERBB2-3UTR_R: 5′-CATCTAGAGACAAAGTGGGTGTGGAGAA-3′.

### Quantitative RT-PCR

Mature miRNAs expression was evaluated by Taqman miRNA assays (Applied Biosystem) specific for miR-125b (miR-125b-5b in miRBase release 19) and RNU6B as reference gene according to the manufacturer’s protocol. Briefly, 5 ng of total RNA was reverse transcribed using the specific looped primer; reverse transcription quantitative PCR was conducted using the standard Taqman miRNA assay protocol on a Biorad-Chromo4 thermal cycler. EPO (assay ID Hs01071097_m1) and EPOR (assay ID Hs00959427_m1) gene expression was assessed by RT-qPCR using Taqman gene expression assays (Applied Biosystem) according to manufacturer’s protocol. 18S was used as reference gene and its expression was assessed using the following primers F: 5′- CTGCCCTATCAACTTTCGATGGTAG-3′; R: 5′- CCGTTTCTCAGGCTCCCTCTC-3′ and KAPA SYBR FAST master mix (Kapa Biosystems). Each sample was analyzed in triplicate. The level of each miRNA/gene was measured using Cq (threshold cycle). The amount of target, normalized on reference gene, was calculated using 2^-ΔCq^ (Comparative Cq) method. Graphs and statistical analyses were performed using GraphPad Prism 5 software.

### Microarray and data analysis

Four samples derived from MCF7 transfection with miR-125b at 24 and 48 hrs were used for microarray analysis. Global gene expression was detected using Agilent Whole Human Genome microarray (#G4112F, Agilent Technologies), which represent 41,000 unique human transcripts. About 500 ngs of total RNA were employed in each experiment. RNA labeling and hybridization were performed in accordance to manufacturer’s indications. Agilent scanner and the Feature Extraction software v.10.5 (Agilent Technologies) were used to obtain the microarray raw-data. Raw data have been submitted to ArrayExpress with the following accession number: E-MTAB-1604. Microarray results were analysed using the GeneSpring GX 12 software (Agilent Technologies, Palo Alto, CA). Data transformation was applied to set all negative raw values at 1.0, followed by a normalization on 75^th^ percentile. A filter on low gene expression was used to keep only the probes expressed in at least one sample. Genes were ordered from the most down-regulated at 24 and 48hrs compared to controls to the most up-regulated and used for sylamer analysis.

### Pathway analysis

Functional Ontology Enrichment analysis and Pathway map visualization was performed using MetaCore pathway analysis by GeneGo (GeneGo Inc.).

### Sylamer analysis

Sylamer analysis was performed through the web-interface Sylarray (http://www.ebi.ac.uk/enright-srv/sylarray) to detect miR-125b target sequence in 3′ untranslated regions (3′UTR) from a ranked gene list, sorted from downregulated to upregulated in MCF7 + miR-125b at 24 and 48 hours compared to negative controls, as described in [28]. A significant enrichment and/or depletion of 7–8 nts sequences complementary to the miRNAs seeds in the 3′UTR of ordered mRNAs was calculated using hypergeometric statistic.

### Statistical analyses

Statistical analyses were performed using GraphPad Prism 5 software. Two-tailed Mann Whitney and unpaired t-test, with or without Welch’s correction, were used for statistical comparisons as specified in the text, according to data distribution. Correlations were calculated using log2 transformed data and Spearman r correlation (two-tailed p-value).

## Competing interest

The authors disclose no competing interest.

## Authors’ contributions

MN and MF conceived and designed the experiments. MF performed experiments and analyzed data. CB performed bioinformatics analyses. SP carried out luciferase experiments. BZ performed microarray experiments. EC, FC, GM, LL, and LD carried out quantitative PCR and participated in ERBB2 sponge experiment. SV participated in the statistical analysis. MP and PQ contributed samples and clinical information. MF and MN wrote the paper. All authors read and approved the final manuscript.

## Supplementary Material

Additional file 1: Figure S1 miR-125b seed is enriched in down-regulated probes. Sylamer analysis of down-regulated mRNAs revealed a significant enrichment in genes whose 3′UTR contain a miR-125b response element (7-mers and 8-mers seed sequences) and are down-regulated after miR-125b transfection (ordered from the most down-regulated to the less). Click here for file

Additional file 2: Table S1 List of 315 genes down-regulated after miR-125b transfection and potential targets of the miRNA. Click here for file

Additional file 3: Figure S2 EPO/EPOR::miR-125b binding sites. Binding sites of miR-125b in EPO and EPOR 3′UTR detected by DIANA MicroT tools (http://diana.cslab.ece.ntua.gr/microT/). Click here for file

Additional file 4: Figure S3 Down-regulation of EPO and EPOR upon the transfection of miR-125b.The expression levels of EPO (A), EPOR (B) was measured in MCF7 breast cancer cell line after 24 and 48 hours from transfection of miR-125b, by RT-qPCR. 18S was used as reference gene and 2-deltaCq method was used for relative expression calculation. The extent of reduction is referred to MCF7 transfected with the scramble (P < .001 for both time points). Each experiment was performed in triplicate. Click here for file

Additional file 5: Table S2 Normalized gene expression data of EPOR and ERBB2 probes, spotted on Agilent microarray, in 69 breast cancers. Click here for file

Additional file 6: Figure S4 Expression of ERBB2 in breast cancer cell lines. MDA-MB-157 and MDA-MB-453 show null or moderate levels of ERBB2 expression, respectively, if compared to MDA-MB-231 (triple negative cell line) and to SK-BR3 (HER2/ERBB2-amplified cell line). The normalized expression was calculated by RT-qPCR using 18S as reference gene; 2-deltaCq method was used for relative expression calculation. Each experiment was performed in triplicate. Click here for file
