# Supplementary material for: miR-125b targets erythropoietin and its receptor and their expression correlates with metastatic potential and ERBB2/HER2 expression
Source: Mol Cancer. 2013 Oct 28;12:130. doi: 10.1186/1476-4598-12-130 (PMC4176119; doi:10.1186/1476-4598-12-130)
Supplement: Additional file 1: Figure S1 — miR-125b seed is enriched in down-regulated probes. Sylamer analysis of down-regulated mRNAs revealed a significant enrichment in genes whose 3′UTR contain a miR-125b response element (7-mers and 8-mers seed sequences) and are down-regulated after miR-125b transfection (ordered from the most down-regulated to the less). [file 1476-4598-12-130-S1.pptx]

## Slide 1
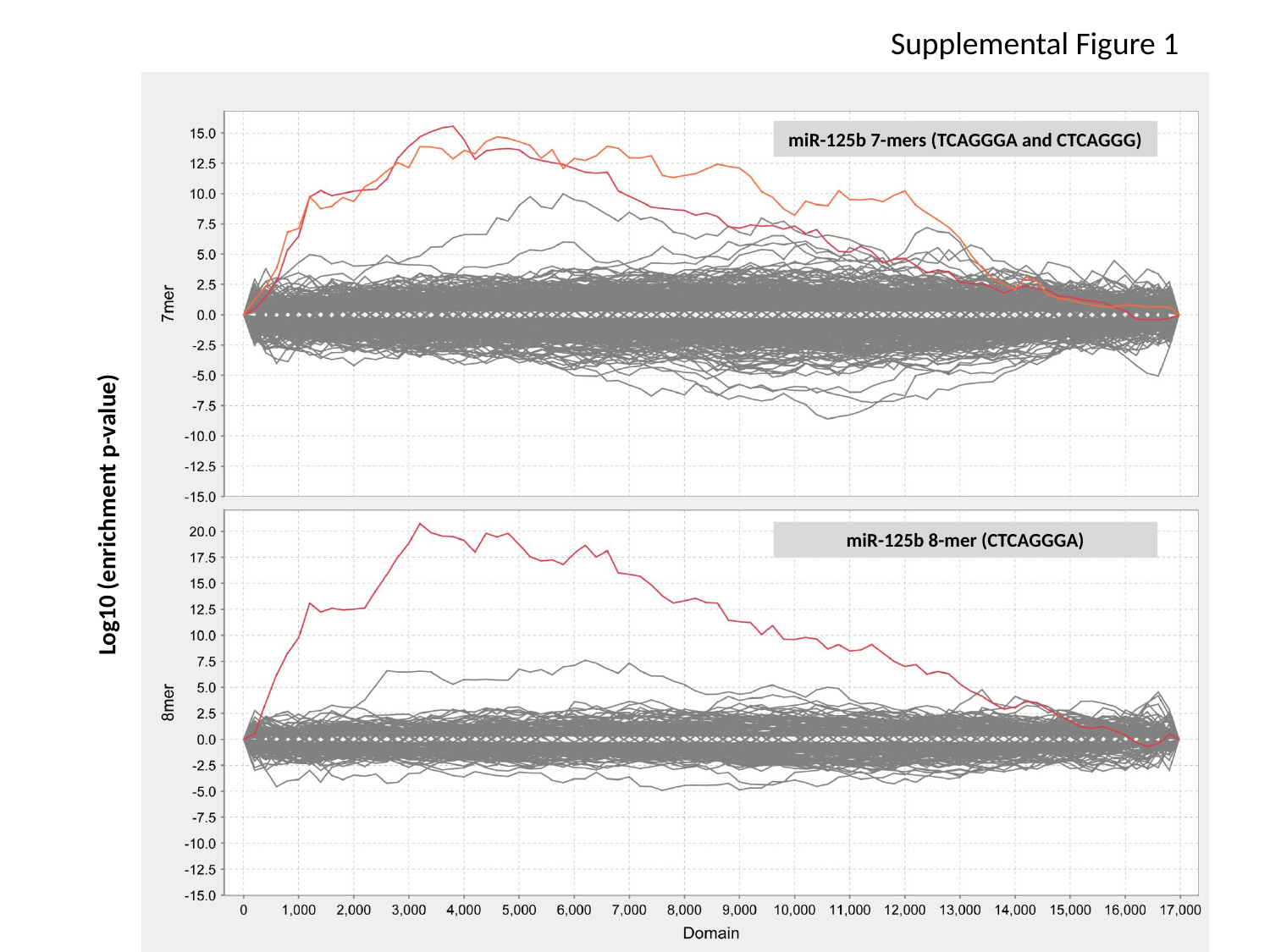

Supplemental Figure 1
miR-125b 7-mers (TCAGGGA and CTCAGGG)
Log10 (enrichment p-value)
miR-125b 8-mer (CTCAGGGA)
