# Supplementary figures and images for: miR-125b targets erythropoietin and its receptor and their expression correlates with metastatic potential and ERBB2/HER2 expression
Source: Mol Cancer. 2013 Oct 28;12:130. doi: 10.1186/1476-4598-12-130 (PMC4176119; doi:10.1186/1476-4598-12-130)

## Slide 1
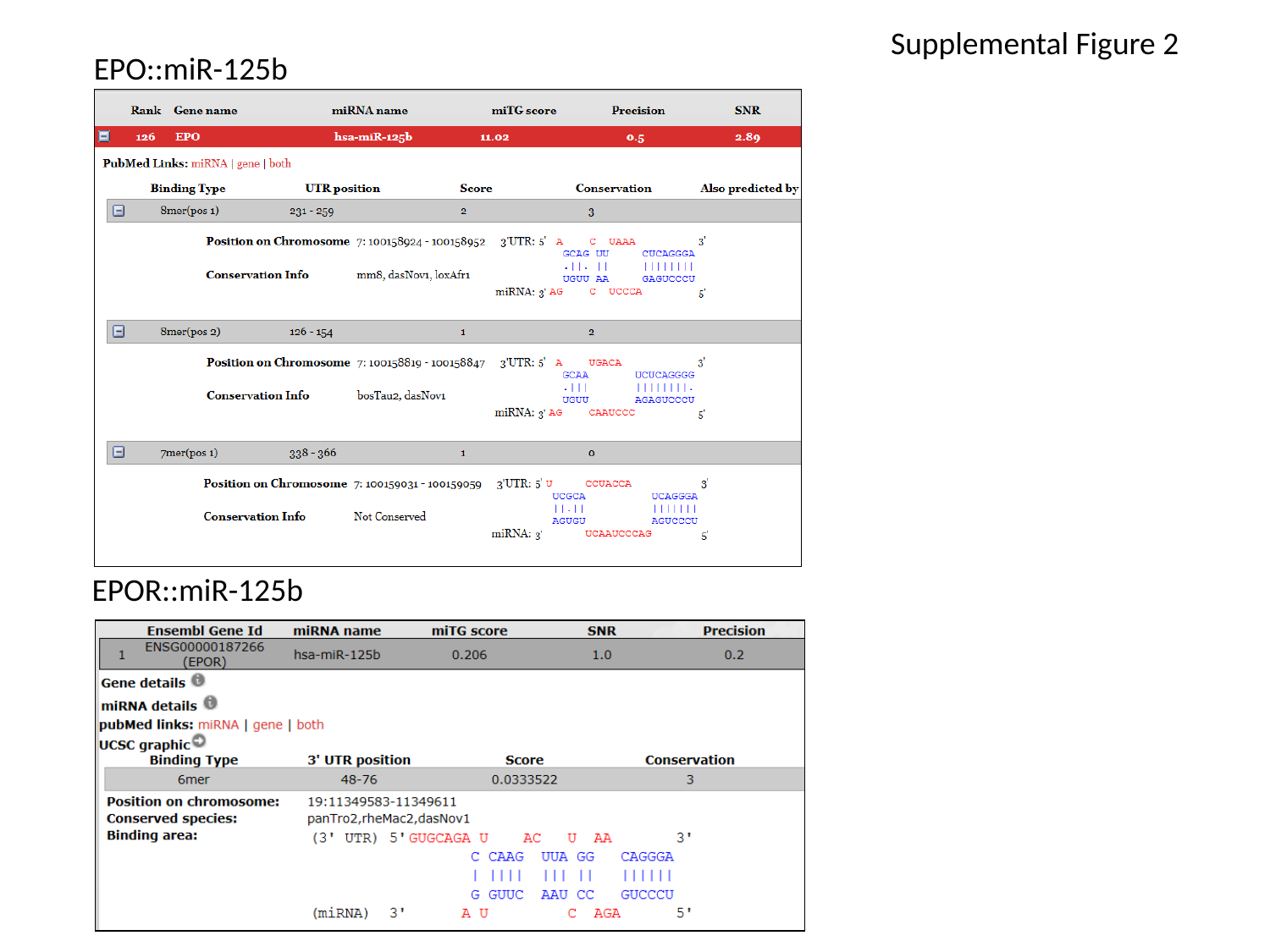

Supplemental Figure 2
EPO::miR-125b
EPOR::miR-125b

Supplement: Additional file 3: Figure S2 — EPO/EPOR::miR-125b binding sites. Binding sites of miR-125b in EPO and EPOR 3′UTR detected by DIANA MicroT tools (http://diana.cslab.ece.ntua.gr/microT/). [file 1476-4598-12-130-S3.pptx]

## Slide 1
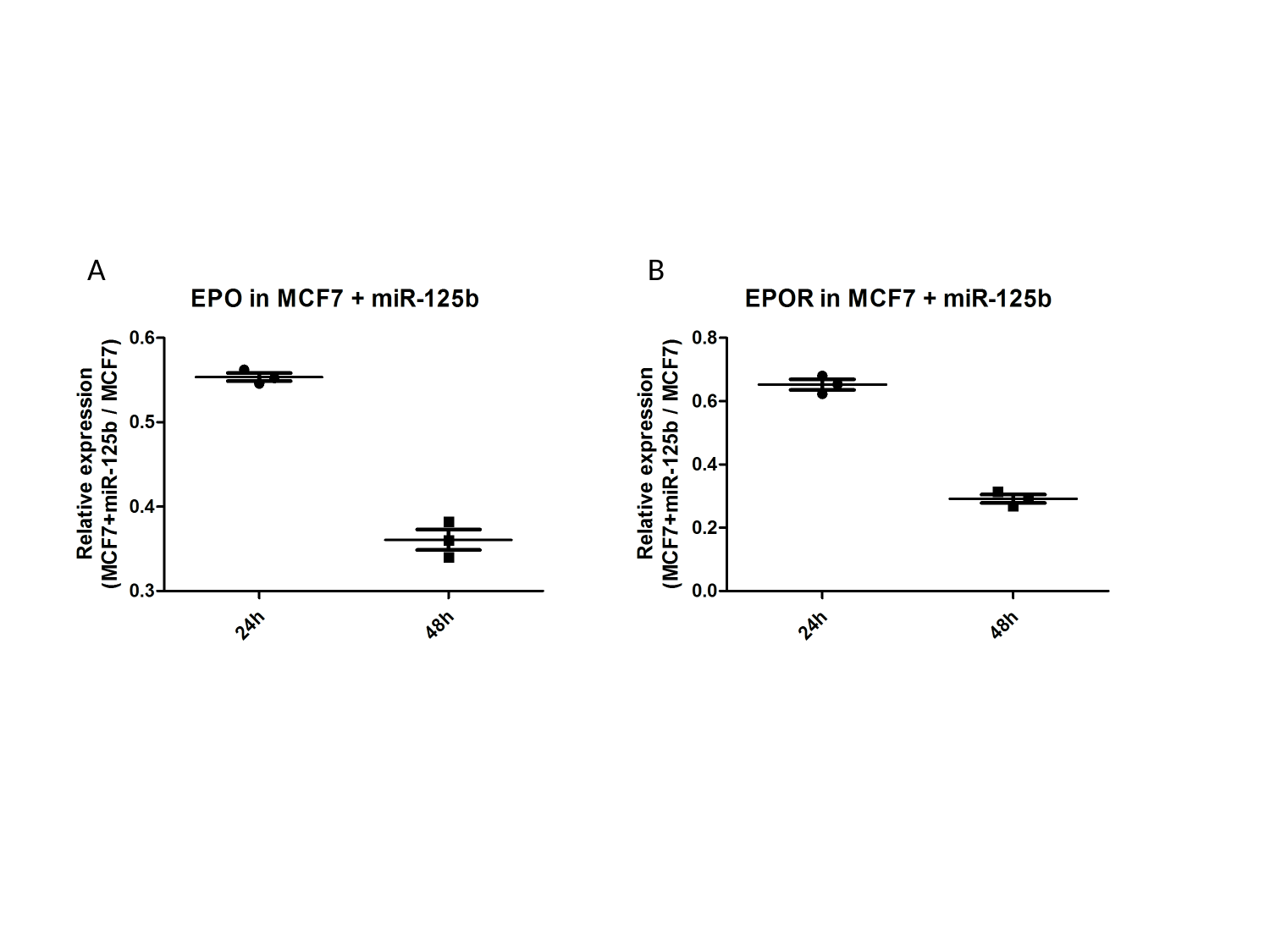

A
B

Supplement: Additional file 4: Figure S3 — Down-regulation of EPO and EPOR upon the transfection of miR-125b.The expression levels of EPO (A), EPOR (B) was measured in MCF7 breast cancer cell line after 24 and 48 hours from transfection of miR-125b, by RT-qPCR. 18S was used as reference gene and 2-deltaCq method was used for relative expression calculation. The extent of reduction is referred to MCF7 transfected with the scramble (P < .001 for both time points). Each experiment was performed in triplicate. [file 1476-4598-12-130-S4.pptx]

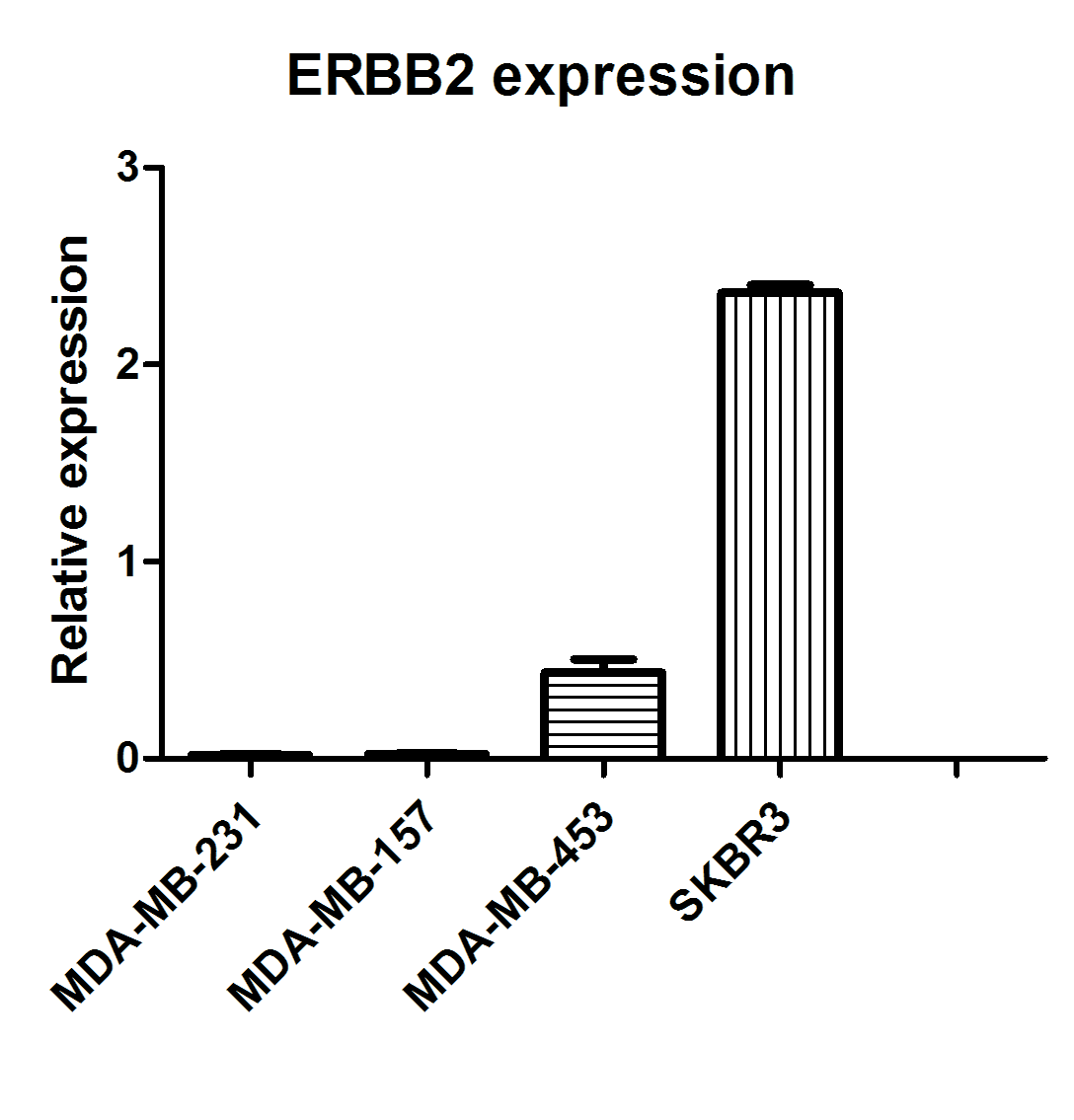

Supplement: Additional file 6: Figure S4 — Expression of ERBB2 in breast cancer cell lines. MDA-MB-157 and MDA-MB-453 show null or moderate levels of ERBB2 expression, respectively, if compared to MDA-MB-231 (triple negative cell line) and to SK-BR3 (HER2/ERBB2-amplified cell line). The normalized expression was calculated by RT-qPCR using 18S as reference gene; 2-deltaCq method was used for relative expression calculation. Each experiment was performed in triplicate. [file 1476-4598-12-130-S6.tiff]
